# Dislocation Transformations at the Common 30°〈0001〉 Grain Boundaries During Plastic Deformation in Magnesium

**DOI:** 10.3390/nano15030232

**Published:** 2025-01-31

**Authors:** Yulong Zhu, Yaowu Sun, An Huang, Fangxi Wang, Peng Chen

**Affiliations:** 1 Key Laboratory of Automobile Materials of Ministry of Education & School of Materials Science and Engineering, Jilin University, Changchun 130025, China; 2 Department of Chemical Engineering, Virginia Tech, Blacksburg, VA 24060, USA

**Keywords:** magnesium alloy, grain boundary, slip transfer, atomistic simulations

## Abstract

After the thermal-mechanical processing of Mg alloys, extensive 30°〈0001〉 grain boundaries (GBs) are present in the recrystallized structure, which strongly affects the mechanical properties via interactions with lattice dislocations. In this work, we systematically investigate how the 30°〈0001〉 GBs influence the slip transmission during plastic deformation. We reveal that basal dislocations can be transmuted into its neighboring grain and continue gliding on the basal plane. The prismatic dislocation can transmit the GB remaining on the same Burgers vector. However, a mobile pyramidal $\langle c+a\rangle$ dislocation can be absorbed at GBs, initiating the formation of new grain. These findings provide a comprehensive understanding on GB-dislocation interaction in hexagonal close-packed (HCP) metals.

## 1. Introduction

Grain boundaries (GBs) are interfaces between two grains with differing crystallographic orientations, and their interaction with dislocations plays a critical role in determining the mechanical properties of structural metals and alloys [1,2]. By controlling and optimizing these interactions through dislocation and boundary characters [3,4], several scenarios may occur: (1) GBs act as source or sink of dislocations to affect dislocation density [5,6]; (2) dislocations can transmit through grain boundaries if the stress is sufficient, depending on the GB orientation, misorientation angle, and dislocation character [7,8]; (3) grain boundaries act as barriers to dislocation motion, leading to increased strength, i.e., the Hall–Petch effect [9,10]; and (4) complicated dislocation reactions may also occur to release the stress accumulation [11,12].

Magnesium (Mg) alloys are the lightest structural materials and have promising applications in the aerospace and automotive industries. However, the mechanical properties need further improvement due to the limited independent slip systems caused by the HCP crystal structure. To enhance the strength and ductility of Mg alloys, their microstructure needs to be engineered towards refined grain size and weakened basal plane texture [13,14]. After thermomechanical processing techniques such as rolling, extrusion and severe plastic deformation, the strong texture can be formed, irrespective of the processing history. The 30°$\langle 0001\rangle$ misorientation has been extensively observed with an intensity peak in the distribution of the misorientation angle in the Rare-earth (RE) and RE-free Mg alloys as well as pure Mg after thermal-mechanical processing [15,16,17].

Thus far, the 30°$\langle 0001\rangle$ GBs have received extensive research interest [18,19]. Dynamic recrystallization leads to a significant weakening of the texture intensity and an increased number of 30° grain boundaries [17,20], with the 30° recrystallized grains persisting throughout the recrystallization process [21,22,23]. Ostapovets et al. [24] suggested that the presence of a peak of 30°$\langle 0001\rangle$ GB peak can be explained by the relatively high frequency of ∑13a and ∑15a, while Li et al. [25] and Wu et al. [26] reported that the 30°$\langle 0001\rangle$ GBs form through grain rotation driven by the dislocation slip. Additionally, Liu and Wang [27] calculated the excess potential energy of the $\langle 0001\rangle$ axis GBs as a function of the misorientation angle and found that the 30°$\langle 0001\rangle$ GBs possess a local minimum energy and are mobile under favorable loading conditions. Moreover, since high volumes of these 30°$\langle 0001\rangle$ GBs always exist in Mg alloys, considerable interactions with lattice dislocations can be expected, resulting in a softening or strengthening effect. However, the interaction mechanisms between dislocations and 30°$\langle 0001\rangle$ GBs during plastic deformation remain unexplored.

The simplest and representative 30° GBs are the 30°$\langle 0001\rangle$ tilt GBs in Mg. Therefore, in this work, we focus on the interactions between 30°$\langle 0001\rangle$ tilt GBs and lattice dislocations on atomic levels using molecular dynamics (MD). The interactions with basal, prismatic, and pyramidal dislocations are systematically investigated for the first time. We reveal that the lattice dislocations can be transmitted to its neighboring grain by complex interaction reactions or absorbed at GB resulting formation of new grains. These findings provide a fresh understanding on dislocation–GB interactions in HCP metals and might offer guidance for grain boundary engineering to achieve improved mechanical properties.

## 2. Method

### 2.1. Experimental Details

The 30°$\langle 0001\rangle$ misorientation was extensively observed with an intensity peak after thermal-mechanical processing such as rolling [15], equal channel angular pressing [24], and fraction stir processing [28]. In this work, we use a low alloyed Mg (0.6Mn-0.4Zn-0.2Ce-0.2Al wt.%) at hand to further examine the misorientation distribution of GBs after hot extrusion. The alloy was prepared from commercial pure Mg (99.85 wt.%), Al (99.90 wt.%), pure Zn (99.90 wt.%) and Mg-5Mn (wt.%), and a Mg−28Ce (wt.%) master alloy. The chemical composition of the experimental alloy was inspected by an optical spectrum analyzer (ARL 4460, Switzerland). The alloy was harmonized at 500 °C for 24 h followed by extrusion at 300 °C with an extrusion ratio of ∼28 and extrusion speed of 0.1 mm/s. The microstructure characterizations were carried out by a Schottky field emission microscope (Zeiss, Sigma 500) equipped with an electron backscatter diffraction (EBSD) detector (Oxford instruments, Symmetry).

### 2.2. Molecular Dynamics Simulations

**Figure 1:**
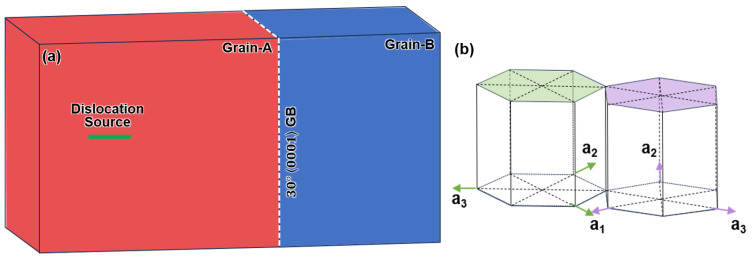
(**a**) Initial schematic for simulating interaction between the lattice dislocations and a 30°$\langle 0001\rangle$ GB. (**b**) The schematic showing the orientation relationship between Grain-A and Grain-B.

Figure 1a displays the bi-crystal model containing the 30°$\langle 0001\rangle$ GB. The orientation relationship between Grain-A and Grain-B is schematically shown in Figure 1b, indicating that the two grains are rotated around the $\langle 0001\rangle$ axis by 15°, respectively. Additionally, to provide more detailed information, we included the relevant model details and crystal orientation in Figures S1–S3 of the Supplementary Materials. The simulation models contain ~300,000 atoms. The simulation system was relaxed using the conjugate gradient algorithm [29], followed by dynamic relaxation for 20 ps in the micro-canonical ensemble (NVE). The samples were then held for an additional 5 ps after the ~10 ps shear step to facilitate the subsequent dislocation generation. The lattice dislocations were introduced into the simulation system by creating a dislocation source [30,31], as shown in Figure 1a. Specifically, we chose two layers of atoms with width of ~5 nm and displaced each layer with respect to each other at a constant speed. As such, dislocations nucleated and glided under the external strain, which was applied on the top surface of the system at a strain rate of ~10^−8^/s.

All MD simulations were performed using the Large-scale Atomic/Molecular Massively Parallel Simulator (LAMMPS) code [32], and EAM interatomic potential [33] for the Mg-Al binary system was employed. This EAM potential was well developed by Liu et al. [34] and has been widely used in numerous atomistic simulations of deformation mechanisms in Mg [35,36]. The open visualization tool (OVITO) [37] was used to visualize the simulation data. Common neighbor analysis (CNA) [38] and dislocation analysis (DXA) [39] were utilized to analyze the character of dislocation and interface.

## 3. Results and Discussion

### 3.1. The 30°$\langle 0001\rangle$ GB

**Figure 2:**
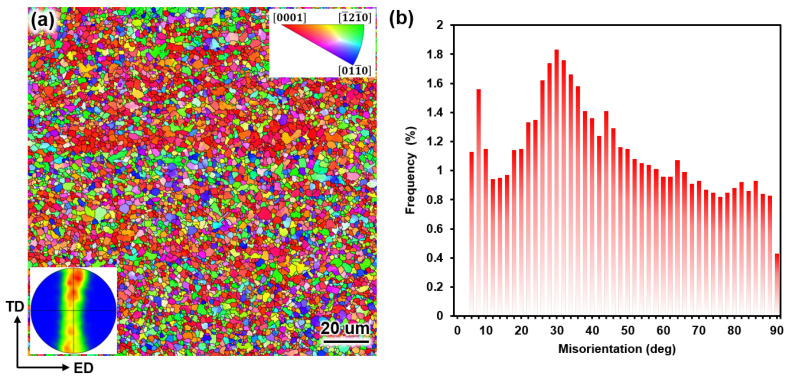
(**a**) The IPF map and (0002)-pole figure of the extruded Mg alloy. (**b**) Distribution of GB misorientation.

The inverse pole map (Figure 2a) of the extruded Mg alloy displays a typical fiber texture with the majority of basal planes of the grains parallel to ED. The distribution of misorientation angles is shown in Figure 2b, with an obvious peak appearing around 30°. This further indicates that understanding the plastic deformation mechanism of the 30°$\langle 0001\rangle$ tilt grain boundaries in Mg alloys should be particularly important. While our current study focuses on a GB misorientation of 30 degrees, we acknowledge the importance of exploring both lower-angle (0–10 degrees) and higher-angle boundaries, as well as their interaction with dislocations, in future research.

**Figure 3:**
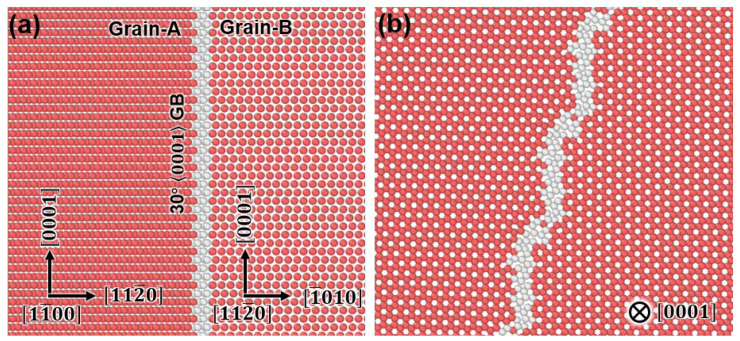
The atomic configuration for the 30°$\langle 0001\rangle$ GB: (**a**) 2D view along the $\left[1\bar{1}00\right]$ of Grain-A. (**b**) Projection view along the $\left[0001\right]$ direction.

Following the experimental observation, we select a representative 30°$\langle 0001\rangle$ GB to perform MD simulations. Figure 3a shows the atomic configuration of the 30°$\langle 0001\rangle$ GB viewed along the $\left[1\bar{1}00\right]$ of the Grain-A, showing the $\left(11\bar{2}0\right)$_A_ ∥ $\left(10\bar{1}0\right)$_B_ interface. Figure 3b indicates that the grain boundary plane displays a periodic structure, consistent with the atomic structure of the common tilt GBs [40,41].

### 3.2. The Interaction Between Basal Dislocations and the 30°$\langle 0001\rangle$ GB

**Figure 4:**
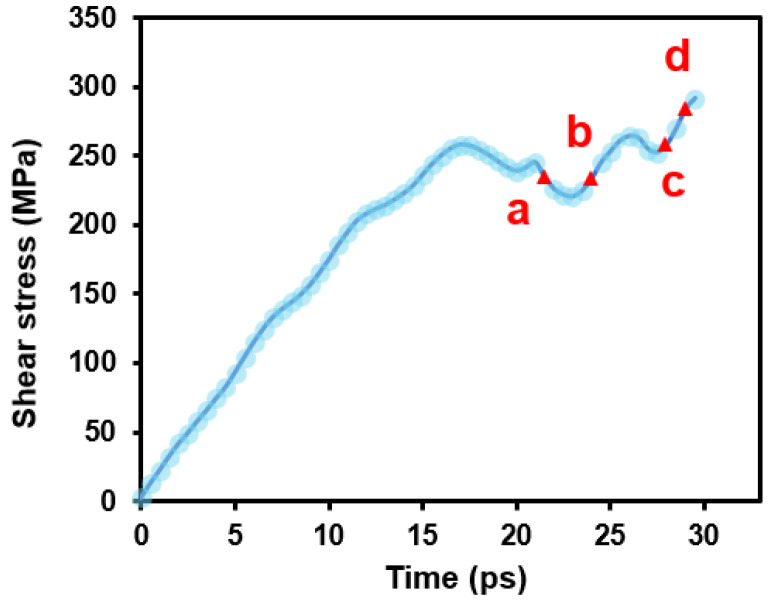
Shear stress evolution during the basal dislocation–GB interaction. The labels denote the corresponding snapshots in Figure 5.

In this section, we simulate how basal dislocations interact with the 30°$\langle 0001\rangle$ GB. Figure 4 shows the stress evolution of the simulation system during deformation. At the beginning, the stress increases monotonically as the deformation proceeds. Once lattice dislocation is nucleated from the dislocation source (Figure 2a), the stress drops rapidly (at ~17 ps). Subsequently, the stress varies as the dislocation glides to and interacts with the GB. A video (Video S1) illustrating the detailed interaction process is included in the Supplemental Material.

**Figure 5:**
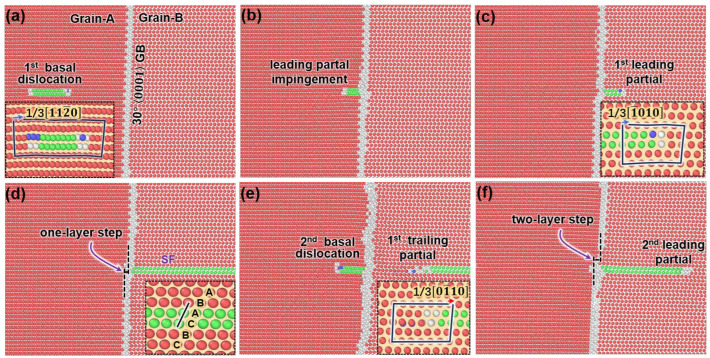
Interaction between the basal dislocations and 30°$\langle 0001\rangle$ GB: (**a**) The basal dislocation glides toward the GB. (**b**) The leading partial dislocation impinges on the GB. (**c**) The trailing partial in Grain-A is absorbed, and a leading partial is nucleated from the GB and glides into Grain-B. (**d**) The leading partial in Grain-B glides away, leaving SF behind. (**e**) The second leading partial in Grain-A impinges on the GB; meanwhile a trailing partial is nucleated in Grain-B. (**f**) The second basal dislocation of Grain-A is fully absorbed at GB, and a new leading partial nucleates from the GB.

In Figure 5a, when the local stress at the dislocation source reaches a critical level, a basal dislocation is nucleated. The inset shows that the Burgers vectors of this basal dislocation are $\frac{1}{3}\left[11\bar{2}0\right]$. Because of the low stacking fault (SF) energy of the basal dislocation, it further dissociates into leading and trailing partial bonding the stacking fault (green atoms) in between, i.e., $\frac{1}{3}\left[11\bar{2}0\right]\to \;\frac{1}{3}\left[01\bar{1}0\right]+SF+\frac{1}{3}\left[10\bar{1}0\right]$. Under the external shear strain, the basal dislocation glides to the right. In Figure 5b, the leading partial has impinged on the GB, while the trailing partial is falling behind, and the SF can still be seen. As the trailing partial is also incorporated into the GB (Figure 5c), a new leading partial is immitted from the GB and glides inside Grain-B to the right. The inset shows that the Burgers vector of the emitted leading partial is $\frac{1}{3}\left[\bar{1}010\right].$ As this leading partial is gliding away, a long two-layer SF is left behind (Figure 5d). As shown in the inset figure, the stacking sequence is changed from “…ABABAB…” to “…ABACBC….”. It can also be seen that a one-layer step is generated at the interface.

If we correlate the above interaction process to the stress curve in Figure 4, the evolution of flow stress now can be understood. When the basal dislocation is nucleated in Grain-A, the stress is decreased (label a). As the leading partial is incorporated into GB, the stress begins increasing (label b). Once the whole dislocation is absorbed and a new leading partial is created, the stress is released again (label c). However, as the leading partial is gliding away, one end of the SF is anchored at the GB; thus, the stress is increased again (label d).

If the external strain is continued, another basal dislocation will be nucleated at the dislocation source in Grain-A and glides to the GB. As shown in Figure 5e, as the leading partial is incorporated into the GB, a trailing partial is nucleated in grain-B and detaches from the GB, resulting in the erasing of SF. The Burgers vector of this trailing partial is determined as $\frac{1}{3}\left[0\bar{1}10\right]$. As the second basal dislocation is fully absorbed by the GB, another leading partial will also be nucleated in Grain-B, and the GB step is increased to two layers (Figure 5f).

Unlike the interactions between the basal dislocations and $\left(10\bar{1}2\right)$ twin boundaries (TB) [42], where the basal dislocation is completely absorbed by the TB and no transmutation occurs, in this work, the basal planes of both grains are in parallel, and there is only misalignment between vectors; thus, the basal-to-basal dislocation transmutation can occur across the GB. However, a full $\frac{1}{3}\left[11\bar{2}0\right]$ basal dislocation in Grain-A can only be transformed to a $\frac{1}{3}\left[\bar{1}010\right]$ leading partial in Grain-B. To generate the trailing partial in Grain-B, another $\frac{1}{3}\left[\bar{1}010\right]$ leading partial of Grain-A is needed. The possible reason may be related to the dislocation reactions with the interface dislocations at the GB.

### 3.3. The Interaction Between Prismatic Dislocations and the 30°$\langle 0001\rangle$ GB

**Figure 6:**
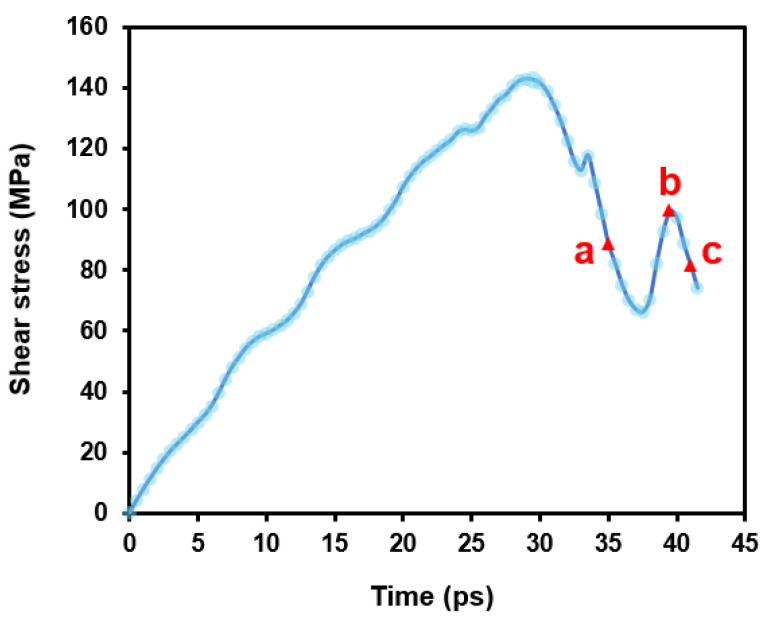
Shear stress evolution of the simulation system during prismatic dislocation–GB interaction. The labels correspond to the snapshots in Figure 7.

**Figure 7:**
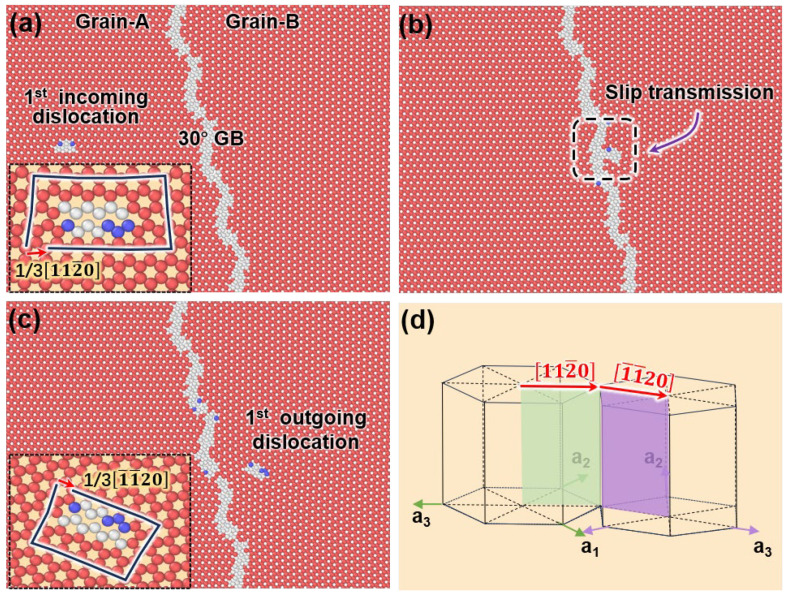
Interaction between the prismatic dislocation and the GB: (**a**) When the shear strain reaches a critical value, a prismatic dislocation is nucleated. The inset shows the Burgers vector analysis. (**b**) The prismatic dislocation impinges on the GB, and a defect is nucleated in the neighboring grain. (**c**) The transmitted dislocation detaches the GB. (**d**) The crystallographic relationship between the two grains. The prismatic systems (slip planes and vectors) in each grain are indicated by colored planes and arrows.

Figure 6 shows the stress evolution of the simulation system during the interaction between prismatic dislocations and 30°$\langle 0001\rangle$ GB. The red labels correspond to the snapshots displayed in Figure 7. Initially, the system was elastically deformed, and the stress increases almost linearly. A sudden drop in flow stress occurs due to the nucleation of prismatic dislocation. As the prismatic dislocation impinges on the GB and is transmitted into its neighboring grain, the stress will increase first and then decrease. A video (Video S2) illustrating the detailed interaction process is included in the Supplemental Material.

In Figure 7a, it can be determined that the nucleated dislocation is on the prismatic plane with a Burgers vector of $\frac{1}{3}\left[11\bar{2}0\right]$. Under the shear strain, the prismatic dislocation glides to the GB, and, as it is transmitting the GB (Figure 7b), the flow stress is increased in the stress curve. Meanwhile, a substructure is nucleated in the neighboring grain. In Figure 7c, this structure detaches from the GB and becomes a dislocation inside Grain-B. After careful analysis, the dislocation is also identified as prismatic dislocation with a Burgers vector of $\frac{1}{3}\left[\bar{1}\bar{1}20\right]$. It is also noted that the flow stress decreases again. Similar to the interaction between lattice dislocation and the twin [43], when the outing dislocation remains highly mobile, it may contribute to strain hardening without sacrificing ductility.

In Figure 7d, a crystallographic relationship between the two prismatic slip systems of two grains is displayed. It can be found that the disorientation angles for the slip plane and Burgers vector in the neighboring grains before and after interaction are 30°. Thus, the hardening effect mostly resulted from the misalignment between the slip systems. There are also some criteria proposed to predict the dislocation transmission across GBs [44,45,46,47,48], which mainly consider a series of geometric characters, including the angle between slip directions and the angle between the slip plane traces on the GB plane, etc. Therefore, our atomistic simulations can be used to validate these criteria.

### 3.4. The Interaction Between Pyramidal Dislocation and 30°$\langle 0001\rangle$ GB

**Figure 8:**
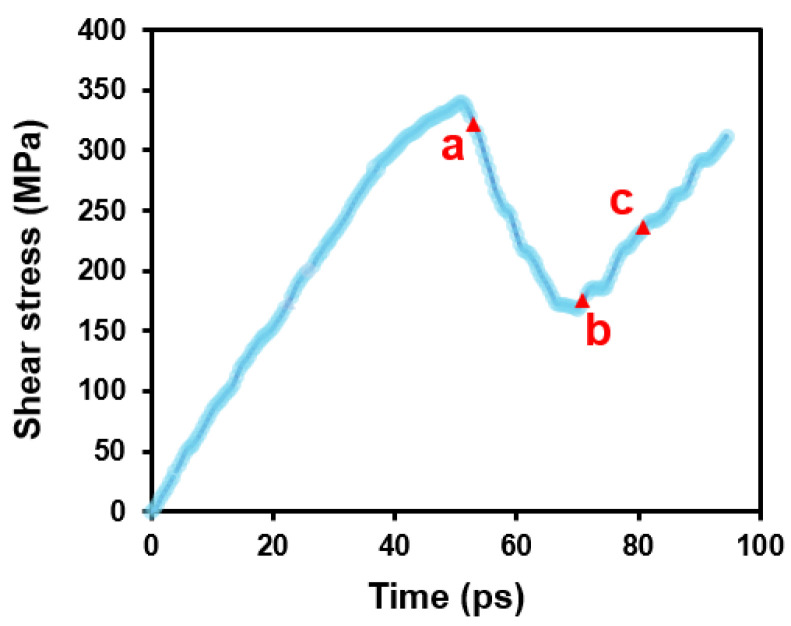
Shear stress evolution during the interaction between GB and pyramidal dislocation. The red labels correspond to the snapshots displayed in Figure 9a–c.

**Figure 9:**
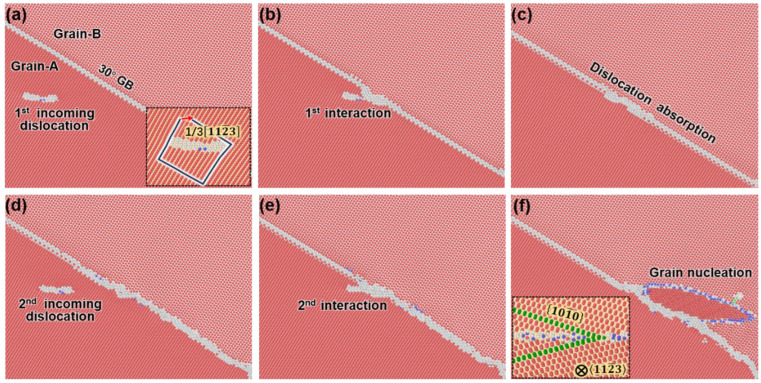
Snapshots in time sequence showing the pyramidal dislocation glides and interacting with the GB: (**a**) The pyramidal dislocation glides toward the GB. (**b**) The pyramidal dislocation impinges on the GB. (**c**) The pyramidal dislocation is completely absorbed by the GB. (**d**) The second pyramidal dislocation is nucleated and glides toward the GB. (**e**) The second pyramidal dislocation impinges on the GB. (**f**) A new grain is nucleated.

Similarly, the pyramidal dislocations are also generated by introducing a dislocation source. The flow stress evolution during the interaction between pyramidal dislocations and 30°$\langle 0001\rangle$ GB is displayed in Figure 8. Once the local stress at the dislocation source reaches a critical value, a pyramidal dislocation will appear, immediately leading to a decrease in the flow stress. As displayed in Figure 9a, this pyramidal dislocation will slip to the GB under the shear deformation. The inset is an edge-on view of the pyramidal dislocation, illustrating that its slip plane is $\left\{11\bar{2}2\right\}$ and its Burgers vector is $\frac{1}{3}\left[11\bar{2}3\right]$, i.e., $\langle c+a\rangle$. As the pyramidal dislocation is gliding to the GB, the flow stress continues decreasing. However, as it is incorporated into the GB (Figure 9b), the flow stress increases dramatically. As the deformation further proceeds, the pyramidal dislocation is completely absorbed by the GB (Figure 9c). At the intersection, the GB structure becomes less coherent, and no dislocation transmutation or transmission occurs. A video (Video S3) illustrating the detailed interaction process has been included in the Supplemental Material.

However, another pyramidal dislocation can be nucleated if the shear strain is continued (Figure 9d). Surprisingly, as this dislocation is absorbed at the GB, a new grain is generated inside the neighboring grain (Figure 9f). This phenomenon is very similar to the formation of new grains through recrystallization.

**Figure 10:**
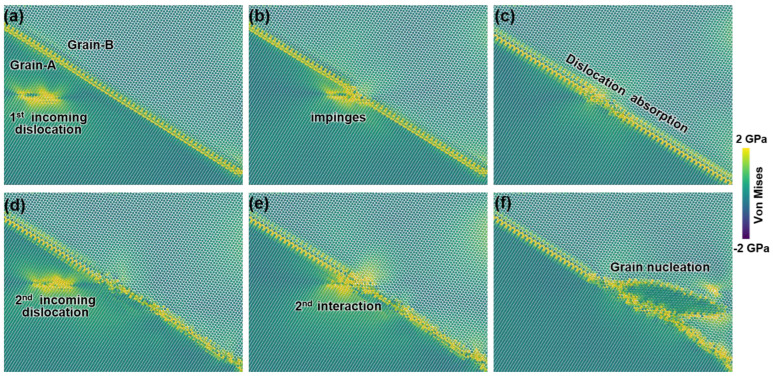
The corresponding von Mises stress field of the snapshots depicted in Figure 9. (**a**) Pyramidal dislocation glides to GB. (**b**) Pyramidal dislocation impinges on the GB. (**c**) Pyramidal dislocation absorbed by GB. (**d**) Second pyramidal dislocation nucleates and glides to GB. (**e**) Second pyramidal dislocation impinges on the GB. (**f**) New grain nucleated.

To further understand the initiation of graining, we calculate the von Mises stress [49], as shown in Figure 10. Obviously, as the pyramidal dislocation is absorbed by the GB, high local stress is accumulated at the GB (Figure 10e). The stress concentration leads to atomic distortion at the interface, providing sites for the subsequent nucleation of new grain or twin [50,51,52]. Therefore, a new grain is nucleated (Figure 9f), resulting in the release of local stress and forming a strain-free zone inside the new grain (Figure 10f).

It has been recognized that the $\langle c+a\rangle$ dislocation is energetically unstable and prone to dissociate at the basal plane, becoming a sessile dislocation and damaging the ductility of Mg [53]. In this work, the pyramidal $\langle c+a\rangle$ dislocation is highly mobile because the stress of the system is sufficiently high, consistent with the in situ TEM observation [54]. In bulk material, as revealed by TEM observations, mobile pyramidal $\langle c+a\rangle$ dislocation can be nucleated at stress concentrators, such as crystal defects like GBs [55,56,57,58]. However, as demonstrated in this work, these mobile pyramidal dislocations cannot transmit but are absorbed at the GBs; thus, the density of mobile $\langle c+a\rangle$ dislocations is reduced and cannot mediate the local strain along the $\langle c\rangle$-axis of grains, deteriorating the ductility and initiating cracks. Therefore, we reveal another possible reason for the limited plasticity of Mg from the perspective of the pyramidal dislocation–GB interactions.

## 4. Conclusions

30°$\langle 0001\rangle$ GBs are extensively generated in Mg alloys after thermal-mechanical processing due to recrystallization. In this work, the 30° GBs are observed with high frequency due to recrystallization. We systematically investigated the interactions between lattice dislocations and this GB by atomistic simulations. The following conclusions can be reached:

Crystallographically, the basal planes of the neighboring grains on each side of the 30°$\langle 0001\rangle$ GB are in parallel. Therefore, a basal dislocation can be transmuted to a basal dislocation in its neighboring grain, but complex reactions between the partials are involved.

A prismatic dislocation can transmit the 30°$\langle 0001\rangle$ GB and become a prismatic dislocation in its neighboring grain. However, the misalignment angles for both the slip plane and Burgers vector of the incoming and outgoing dislocations are 30°; thus, a strong hardening effect may be induced during such interactions.

The mobile pyramidal $\langle c+a\rangle$ dislocation cannot be transmuted into its neighboring grain. Instead, these dislocations are absorbed by the GB, which acts as a dislocation sink.

The dislocation–GB interactions, including slip transfer and absorption, in HCP metals, offer valuable insights for GB engineering to improve mechanical properties.

## Data Availability

The data that support the findings of this study are available from the corresponding author upon reasonable request.

## Supplementary Materials

The following supporting information can be downloaded at https://www.mdpi.com/article/10.3390/nano15030232/s1, Figure S1: Initial configuration for simulating interaction between the basal dislocations and a 30°$\langle 0001\rangle$ GB. Figure S2: Initial configuration for simulating interaction between the prismatic dislocations and a 30°$\langle 0001\rangle$ GB. Figure S3: Initial configuration for simulating interaction between the pyramidal dislocations and a 30°$\langle 0001\rangle$ GB. Video S1: Interaction between the basal dislocations and the 30°$\langle 0001\rangle$ GB. Video S2: Interaction between the prismatic dislocation and the GB. Video S3: The pyramidal dislocation interacts with the GB.

## References

[1] Huang Q., Yu D., Xu B., Hu W., Ma Y., Wang Y., Zhao Z., Wen B., He J., Liu Z. (2014). Nanotwinned Diamond with Unprecedented Hardness and Stability. Nature.

[2] Hou J.X., Liu S.F., Cao B.X., Luan J.H., Zhao Y.L., Chen Z., Zhang Q., Liu X.J., Liu C.T., Kai J.J. (2022). Designing Nanoparticles-Strengthened High-Entropy Alloys with Simultaneously Enhanced Strength-Ductility Synergy at Both Room and Elevated Temperatures. Acta Mater..

[3] McMurtrey M.D., Was G.S., Patrick L., Farkas D. (2011). Relationship between Localized Strain and Irradiation Assisted Stress Corrosion Cracking in an Austenitic Alloy. Mater. Sci. Eng. A.

[4] Jiao Z., Was G.S. (2011). Impact of Localized Deformation on IASCC in Austenitic Stainless Steels. J. Nucl. Mater..

[5] Hugo R.C., Kung H., Weertman J.R., Mitra R., Knapp J.A., Follstaedt D.M. (2003). In-Situ TEM Tensile Testing of DC Magnetron Sputtered and Pulsed Laser Deposited Ni Thin Films. Acta Mater..

[6] Kumar K.S., Suresh S., Chisholm M.F., Horton J.A., Wang P. (2003). Deformation of Electrodeposited Nanocrystalline Nickel. Acta Mater..

[7] De Koning M., Kurtz R.J., Bulatov V.V., Henager C.H., Hoagland R.G., Cai W., Nomura M. (2003). Modeling of Dislocation–Grain Boundary Interactions in FCC Metals. J. Nucl. Mater..

[8] Jang H., Farkas D. (2007). Interaction of Lattice Dislocations with a Grain Boundary during Nanoindentation Simulation. Mater. Lett..

[9] Yu H., Xin Y., Wang M., Liu Q. (2018). Hall-Petch Relationship in Mg Alloys: A Review. J. Mater. Sci. Technol..

[10] Cordero Z.C., Knight B.E., Schuh C.A. (2016). Six Decades of the Hall–Petch Effect—A Survey of Grain-Size Strengthening Studies on Pure Metals. Int. Mater. Rev..

[11] Jin Z.-H., Gumbsch P., Albe K., Ma E., Lu K., Gleiter H., Hahn H. (2008). Interactions between Non-Screw Lattice Dislocations and Coherent Twin Boundaries in Face-Centered Cubic Metals. Acta Mater..

[12] Li N., Wang J., Huang J.Y., Misra A., Zhang X. (2010). In Situ TEM Observations of Room Temperature Dislocation Climb at Interfaces in Nanolayered al/Nb Composites. Scr. Mater..

[13] Chino Y., Sassa K., Kamiya A., Mabuchi M. (2006). Enhanced Formability at Elevated Temperature of a Cross-Rolled Magnesium Alloy Sheet. Mater. Sci. Eng. A.

[14] Handbook A.S. (1999). Magnesium and Magnesium Alloys. ASM international.

[15] Basu I., Pradeep K.G., Mießen C., Barrales-Mora L.A., Al-Samman T. (2016). The Role of Atomic Scale Segregation in Designing Highly Ductile Magnesium Alloys. Acta Mater..

[16] Steiner M.A., Bhattacharyya J.J., Agnew S.R. (2015). The Origin and Enhancement of {0001} <$11\bar{2}0$> Texture during Heat Treatment of Rolled AZ31B Magnesium Alloys. Acta Mater..

[17] Molodov K.D., Al-Samman T., Molodov D.A., Gottstein G. (2014). Mechanisms of Exceptional Ductility of Magnesium Single Crystal during Deformation at Room Temperature: Multiple Twinning and Dynamic Recrystallization. Acta Mater..

[18] Zhang S., Xie Z., Keuter P., Saood S., Abdellaoui L., Zhou X., Cautaerts N., Breitbach B., Aliramaji S., Korte-Kerzel S. (2022). Atomistic Structures of <0001> Tilt Grain Boundaries in a Textured Mg Thin Film. Nanoscale.

[19] Huber L., Rottler J., Militzer M. (2014). Atomistic Simulations of the Interaction of Alloying Elements with Grain Boundaries in Mg. Acta Mater..

[20] Panigrahi S.K., Yuan W., Mishra R.S., DeLorme R., Davis B., Howell R.A., Cho K. (2011). A Study on the Combined Effect of Forging and Aging in Mg–Y–RE Alloy. Mater. Sci. Eng. A.

[21] Bhattacharyya J.J., Agnew S.R., Muralidharan G. (2015). Texture Enhancement during Grain Growth of Magnesium Alloy AZ31B. Acta Mater..

[22] Barrett C.D., Imandoust A., Oppedal A.L., Inal K., Tschopp M.A., El Kadiri H. (2017). Effect of Grain Boundaries on Texture Formation during Dynamic Recrystallization of Magnesium Alloys. Acta Mater..

[23] Gottstein G., Al Samman T. (2005). Texture Development in Pure Mg and Mg Alloy AZ31. MSF.

[24] Ostapovets A., Šedá P., Jäger A., Lejček P. (2011). Characteristics of Coincident Site Lattice Grain Boundaries Developed during Equal Channel Angular Pressing of Magnesium Single Crystals. Scr. Mater..

[25] Li B., Liao M., Ma Q., McClelland Z. (2015). Structure of Grain Boundaries with 30°[0001] Misorientation in Dynamically Recrystallized Magnesium Alloys. Comput. Mater. Sci..

[26] Wu B.L., Wan G., Zhang Y.D., Du X.H., Wagner F., Esling C. (2010). Fragmentation of Large Grains in AZ31 Magnesium Alloy during ECAE via Route a. Mater. Sci. Eng. A.

[27] Liu X., Wang J. (2016). Low-Energy, Mobile Grain Boundaries in Magnesium. Sci. Rep..

[28] Wang W., Han P., Peng P., Zhang T., Liu Q., Yuan S.-N., Huang L.-Y., Yu H.-L., Qiao K., Wang K.-S. (2020). Friction Stir Processing of Magnesium Alloys: A Review. Acta Metall. Sin. (Engl. Lett.).

[29] Bitzek E., Koskinen P., Gähler F., Moseler M., Gumbsch P. (2006). Structural Relaxation Made Simple. Phys. Rev. Lett..

[30] Garg P., Rupert T.J. (2023). Local Structural Ordering Determines the Mechanical Damage Tolerance of Amorphous Grain Boundary Complexions. Scr. Mater..

[31] Pan Z., Rupert T.J. (2014). Damage Nucleation from Repeated Dislocation Absorption at a Grain Boundary. Comput. Mater. Sci..

[32] Plimpton S. (1995). Fast Parallel Algorithms for Short- Range Molecular Dynamics. J. Comput. Phys..

[33] Baskes M.I., Nelson J.S., Wright A.F. (1989). Semiempirical Modified Embedded-Atom Potentials for Silicon and Germanium. Phys. Rev. B Condens. Matter.

[34] Liu X.Y., Adams J.B., Ercolessi F., Moriarty J.A. (1996). EAM Potential for Magnesium from Quantum Mechanical Forces. Model. Simul. Mater. Sci. Eng..

[35] Wang F.X., Li B. (2018). Origin of Deflection of Precipitates during Interaction with a Migrating Twin Boundary in Magnesium Alloys. Comput. Mater. Sci..

[36] Li B., En M. (2009). Zonal Dislocations Mediating {$10\bar{1}1$} <$10\bar{1}\bar{2}$> Twinning in Magnesium. Acta Mater..

[37] Stukowski A. (2010). Visualization and Analysis of Atomistic Simulation Data with OVITO-the Open Visualization Tool. Model. Simul. Mater. Sci. Eng..

[38] Honeycutt J.D., Andersen H.C. (1987). Molecular Dynamics Study of Melting and Freezing of Small Lennard-Jones Clusters. J. Phys. Chem..

[39] Stukowski A. (2020). Dislocation Analysis Tool for Atomistic Simulations. Handbook of Materials Modeling: Methods: Theory and Modeling.

[40] Khater H.A., Serra A., Pond R.C., Hirth J.P. (2012). The Disconnection Mechanism of Coupled Migration and Shear at Grain Boundaries. Acta Mater..

[41] Sato Y., Roh J.-Y., Ikuhara Y. (2013). Grain-Boundary Structural Transformation Induced by Geometry and Chemistry. Phys. Rev. B.

[42] Chen P., Wang F., Li B. (2019). Dislocation Absorption and Transmutation at {$10\bar{1}2$} Twin Boundaries in Deformation of Magnesium. Acta Mater..

[43] Chen P., Ombogo J., Li B. (2020). Dislocation ↔ Twin Transmutations during Interaction between Prismatic Slip and {$10\bar{1}1$} Twin in Magnesium. Acta Mater..

[44] Ma A., Roters F., Raabe D. (2006). Studying the Effect of Grain Boundaries in Dislocation Density Based Crystal-Plasticity Finite Element Simulations. Int. J. Solids Struct..

[45] Li Z., Hou C., Huang M., Ouyang C. (2009). Strengthening Mechanism in Micro-Polycrystals with Penetrable Grain Boundaries by Discrete Dislocation Dynamics Simulation and Hall–Petch Effect. Comput. Mater. Sci..

[46] Han J., Thomas S.L., Srolovitz D.J. (2018). Grain-Boundary Kinetics: A Unified Approach. Prog. Mater. Sci..

[47] Bieler T.R., Eisenlohr P., Zhang C., Phukan H.J., Crimp M.A. (2014). Grain Boundaries and Interfaces in Slip Transfer. Curr. Opin. Solid. State Mater. Sci..

[48] Clark W.A.T., Wagoner R.H., Shen Z.Y., Lee T.C., Robertson I.M., Birnbaum H.K. (1992). On the Criteria for Slip Transmission across Interfaces in Polycrystals. Scr. Metall. Mater..

[49] Thomas T.Y. (1955). Combined Elastic and von Mises Stress-Strain Relations. Proc. Natl. Acad. Sci. USA.

[50] McCabe R.J., Kumar M.A., Liu W., Tomé C.N., Capolungo L. (2021). Revealing the Effect of Local Stresses on Twin Growth Mechanisms in Titanium Using Synchrotron X-Ray Diffraction. Acta Mater..

[51] Beyerlein I.J., Capolungo L., Marshall P.E., McCabe R.J., Tomé C.N. (2010). Statistical Analyses of Deformation Twinning in Magnesium. Philos. Mag..

[52] Wang J., Beyerlein I.J., Tomé C.N. (2010). An Atomic and Probabilistic Perspective on Twin Nucleation in Mg. Scr. Mater..

[53] Wu Z., Curtin W.A. (2015). The Origins of High Hardening and Low Ductility in Magnesium. Nature.

[54] Liu B.-Y., Liu F., Yang N., Zhai X.-B., Zhang L., Yang Y., Li B., Li J., Ma E., Nie J.-F. (2019). Large Plasticity in Magnesium Mediated by Pyramidal Dislocations. Science.

[55] Jiang L., Gong M., Wang J., Pan Z., Wang X., Zhang D., Wang Y.M., Ciston J., Minor A.M., Xu M. (2022). Visualization and Validation of Twin Nucleation and Early-Stage Growth in Magnesium. Nat. Commun..

[56] Tang Y., El-Awady J.A. (2014). Formation and Slip of Pyramidal Dislocations in Hexagonal Close-Packed Magnesium Single Crystals. Acta Mater..

[57] Wu J., Lu S., Tian J., Chiu Y. (2024). In-Situ TEM Study of Dislocations in Mg–Y Alloys. Mater. Sci. Eng. A.

[58] Gaillard Y., Tromas C., Woirgard J. (2006). Quantitative Analysis of Dislocation Pile-Ups Nucleated during Nanoindentation in MgO. Acta Mater..

