# Supplementary material for: Dislocation Transformations at the Common 30°〈0001〉 Grain Boundaries During Plastic Deformation in Magnesium
Source: Nanomaterials (Basel). 2025 Jan 31;15(3):232. doi: 10.3390/nano15030232 (PMC11819990; doi:10.3390/nano15030232)
Supplement: Supplementary file 1 [file nanomaterials-15-00232-s001.zip › Supplementary Materials figures.pdf]

Dislocation Transformations at the Common 30°⟨0001⟩ Grain Boundaries During Plastic Deformation in Magnesium

Yulong Zhu [1], Yaowu Sun [1], An Huang [1], Fangxi Wang [2,*] and Peng Chen [1,*]

[1] Key Laboratory of Automobile Materials of Ministry of Education & School of Materials Science and Engineering, Jilin University, Changchun 130025, China
[2] Department of Chemical Engineering, Virginia Tech, Blacksburg, VA 24060, USA
* Correspondence: fxwang@vt.edu (F.W.); pchen21@jlu.edu.cn (P.C.)

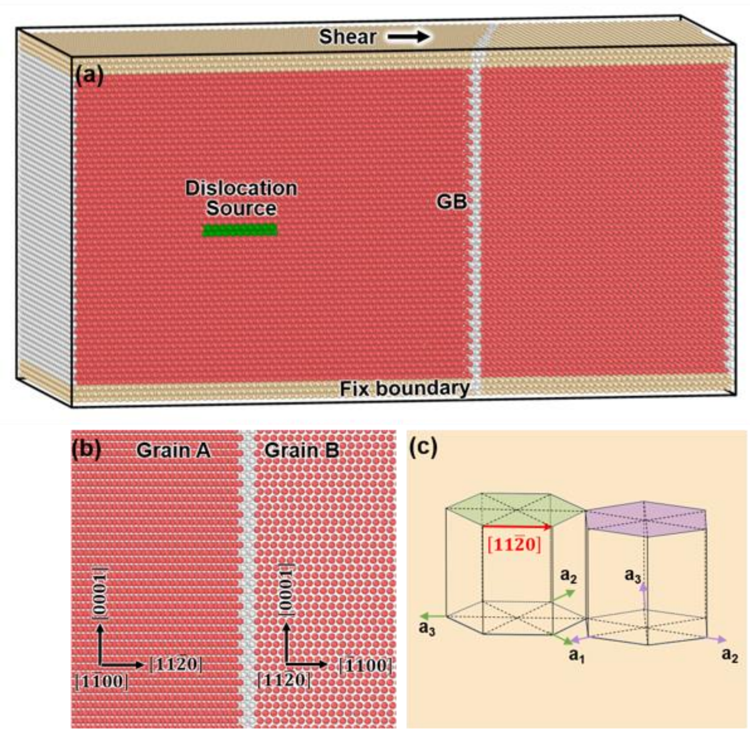

Figure S1. (a) Initial configuration for simulating interaction between the basal dislocations and a 30° ⟨0001⟩ GB. (b) 2D view along the [1$\bar{1}$00] of the grain–A. (c) The schematic showing the orientation relationship between grain-A and grain-B.

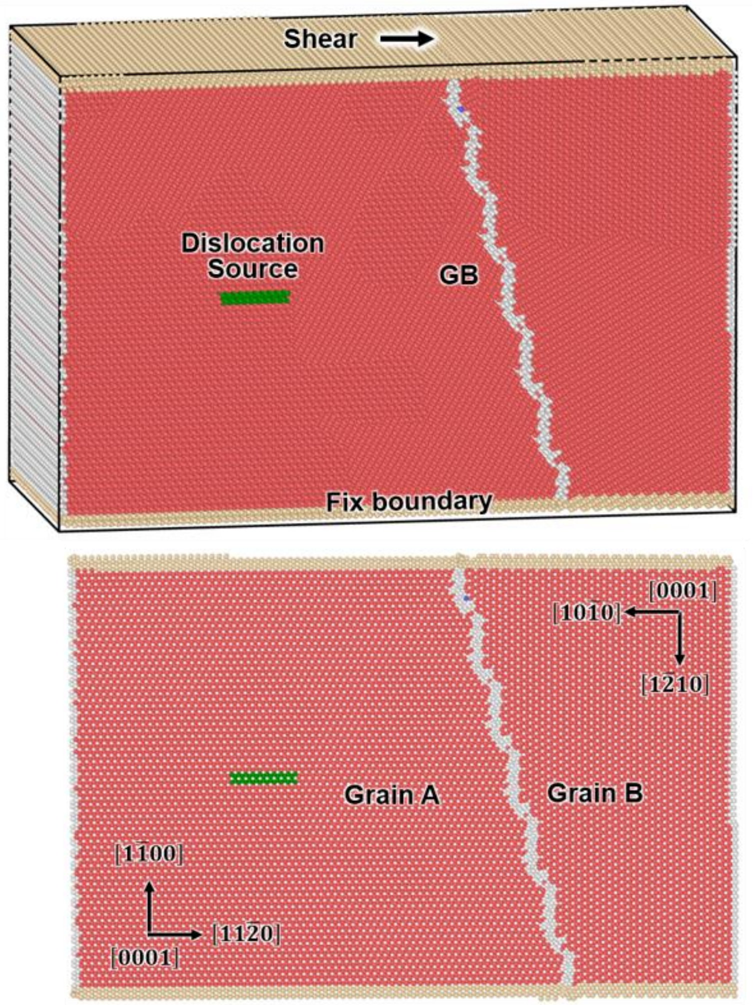

Figure S2. (a) Initial configuration for simulating interaction between the prismatic dislocations and a 30°⟨0001⟩ GB. (b) 2D view along the [0001].

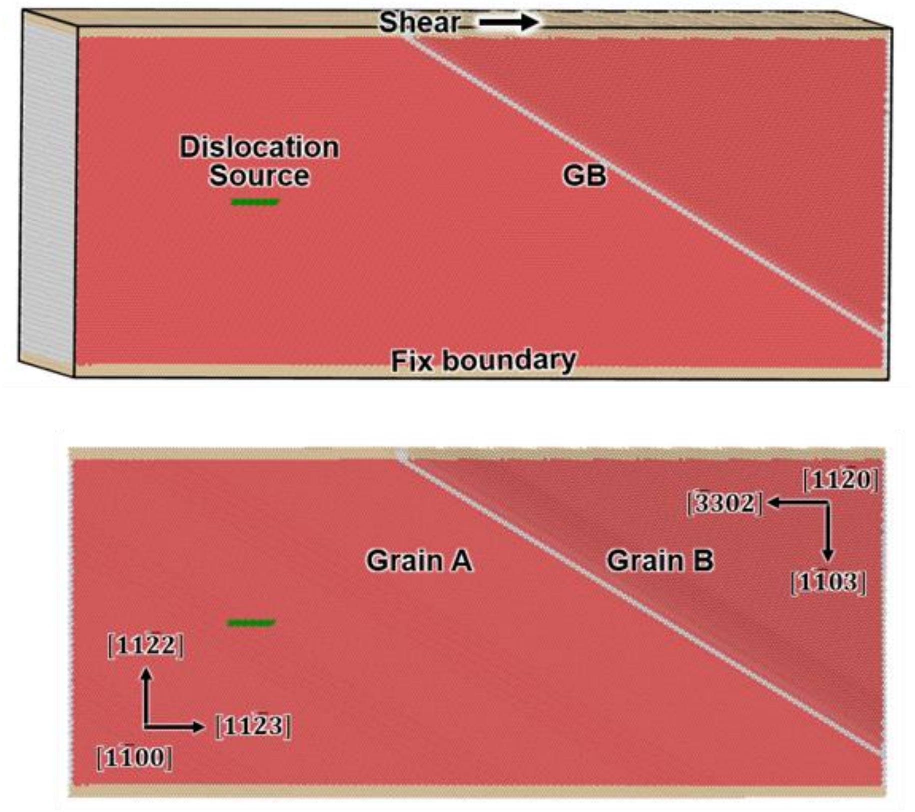

Figure S3. (a) Initial configuration for simulating interaction between the pyramidal dislocations and a 30°⟨0001⟩ GB. (b) 2D view along the $[1\bar{1}00]$ of the grain–A.
